# Exploring the Impact of Digital Peer Support Services on Meeting Unmet Needs Within an Employee Assistance Program: Retrospective Cohort Study

**DOI:** 10.2196/68221

**Published:** 2025-02-25

**Authors:** Harpreet Nagra, Robert A Mines, Zara Dana

**Affiliations:** 1 Supportiv Berkeley, CA United States; 2 MINES & Associates Littleton, CO United States

**Keywords:** digital peer support, peer support, EAPs, cost-effectiveness, SROI

## Abstract

**Background:**

The World Health Organization estimates that 1 in 4 people worldwide will experience a mental disorder in their lifetime, highlighting the need for accessible support.

**Objective:**

This study evaluates the integration of digital peer support (DPS) into an employee assistance program (EAP), testing 3 hypotheses: (1) DPS may be associated with changes in EAP counseling utilization within a 5-session model; (2) DPS users experience reduced sadness, loneliness, and stress; and (3) DPS integration generates a positive social return on investment (SROI).

**Methods:**

The study analyzed EAP utilization within a 5-session model using pre-post analysis, sentiment changes during DPS chats via natural language processing models, and SROI outcomes.

**Results:**

Among 587 DPS chats, 432 (73.6%) occurred after business hours, emphasizing the importance of 24/7 availability. A matched cohort analysis (n=72) showed that DPS reduced therapy sessions by 2.07 per participant (*P*<.001; Cohen d=1.77). Users’ messages were evaluated for sentiments of sadness, loneliness, and stress on a 1-10 scale. Significant reductions were observed: loneliness decreased by 55.04% (6.91 to 3.11), sadness by 57.5% (6.84 to 2.91), and stress by 56.57% (6.78 to 2.95). SROI analysis demonstrated value-to-investment ratios of US $1.66 (loneliness), US $2.50 (stress), and US $2.58 (sadness) per dollar invested.

**Conclusions:**

Integrating DPS into EAPs provides significant benefits, including increased access, improved emotional outcomes, and a high SROI, reinforcing its value within emotional health support ecosystems.

## Introduction

### Background

The World Health Organization estimates that 1 in 4 people globally will experience mental disorders in their lifetime [1], stressing the need for a readily available treatment ecosystem. Relatedly, the increasing popularity of remote work post–COVID-19, along with work- and workplace-related stress—stemming from long hours, high workloads, an inability to disconnect, and, in particular, isolation—has contributed to a rise in mental health problems such as anxiety and depression, musculoskeletal pain, sleep deprivation, and stress [2]. These stressors impact not only employees’ well-being but also presenteeism, absenteeism, and work-life balance [2-4]. Burnout, in particular, arises from chronic stress characterized by emotional, physical, and mental exhaustion, leading to reduced productivity, increased absenteeism, and higher turnover rates [3].

While the Centers for Disease Control and Prevention’s National Center for Health Statistics reports a notable increase in treatment-seeking behaviors between 2019 (19.2%) and 2021 (21.6%), a significant majority of working adults may still suffer alone [5]. The proportion of working adults aged 18-44 years receiving mental health treatment grew significantly from 18.5% in 2019 to 23.2% in 2021 [5], with women (23.8% to 28.6%, respectively) continuing to outpace men (13.1% to 17.8%, respectively) [5]. By 2021, adults aged 45-64 years (21.2%) and those aged 65 years and over (18.9%) ranked second and third, respectively, in seeking treatment [5].

### Employee Assistance Programs

Mental and emotional health–focused employee assistance programs (EAPs), which are confidential, voluntary, organization-sponsored, and low-cost services for employees, offer personal or work-related counseling and support. The purpose of EAPs is to help employees manage stress, enhance problem-solving abilities, and, in turn, improve work productivity, reduce absenteeism, and promote overall well-being [6]. Counseling-based EAPs provide psychoeducation, teach coping strategies, and assist users in setting achievable personal goals that build confidence and self-efficacy [7-12]. Additionally, EAPs offer training in communication skills, such as assertiveness, to improve relationships and help users navigate challenges effectively. Patient activation, which refers to an individual’s knowledge, skills, confidence, and willingness to manage their health and well-being [7,8], is further supported by connecting users to resources and support networks, including health care providers and peer support groups. Finally, EAPs empower users with self-management tools, such as digital apps and resource libraries, that allow them to track their progress and independently manage their well-being [12]. Together, these strategies foster self-reliance, enhancing users’ ability to manage their mental health and overall well-being [7]. Depending on the EAP, a significant return on investment (ROI) may be observed, with some organizations seeing US $3-US $10 for every dollar spent [13], largely due to positive impacts on absenteeism, productivity, and employee turnover and retention [6,13]. EAPs are well-established assets to organizations, delivering emotional benefits to employees and productivity and financial benefits to employers [6,13].

### Digital Peer Support

While counseling-based EAPs are known for their advantageous returns, not every emotional concern requires counseling support. A tiered support system for individuals who are mentally well but experiencing lower-acuity emotional distress may be necessary. Simultaneously, the US health care system currently faces a shortage of mental health professionals, limiting access to adequate support [14,15]. In 2021, 5930 areas were designated as health professional shortage areas, leaving 129.6 million people without access to affordable and accessible care [15]. President Biden’s 2022 White House brief highlighted this issue and called for efforts to “strengthen system capacity,” including the increased use of peer support specialists and paraprofessionals [16].

Peer support interventions involve individuals with similar lived experiences offering mutual support [17]. While expert-based professional support may evoke feelings of shame, failure, and mental health stigma, peer support can normalize emotional struggles and foster connection, validation, and empathy through shared experiences [18]. This “mutual empowerment” from peer-driven interventions grants individuals greater autonomy and competency [18]. However, some Americans may not have access to digital peer services due to a lack of universal internet access and low health literacy. Approximately 24 million people in the United States lack reliable broadband service or high-speed internet [19]. Historically, lower rates of high-speed internet use have been observed in households where the owner is 65 years or older, Hispanic, African American, American Indian, or Alaska Native [19]. Furthermore, in 2021, only about 5 in 10 households with incomes below US $25K used high-speed internet [19]. Additionally, approximately 90 million American adults struggle to understand and navigate complex health- and text-based tasks accurately and consistently, exhibiting low health literacy [20]. By addressing these disparities, digital peer support (DPS) services could become even more inclusive by expanding support to those currently excluded from traditional services.

Self-determination theory, as articulated by Ryan and Deci [21], offers insight into key features that make peer support impactful. The theory states that self-determination and emotional growth are driven by 3 core elements—autonomy, competence, and relatedness [21,22]. Autonomy refers to the ability to self-direct and control one’s actions, competence involves mastering new skills, and relatedness is about forming meaningful connections with others [21,22]. Peer support provides a nonjudgmental, nonstigmatizing space that fosters autonomy, as there is no professional expert directing treatment. Competence is strengthened through the normalization of mistakes shared via lived experiences, as well as the application and practice of coping strategies. Relatedness is reinforced through the verbal and emotional connections formed with similar peers [18]. Research has consistently shown that relatedness in supportive relationships contributes significantly to emotional regulation, motivation, and the overall success of interventions. By reinforcing relatedness through peer-to-peer interactions, peer support programs address a fundamental psychological need that complements autonomy and competence, further validating their essential role in the model [17,18]. The interrelatedness of these dynamic factors is believed to enhance mental health and well-being [17-22]. The introduction of DPS platforms extends these benefits by offering accessible, mutually supportive communities to help alleviate feelings of loneliness and isolation [23]. Numerous studies have demonstrated the effectiveness of these digital interventions in addressing mental health needs [23-30].

Confirmed benefits of peer support interventions are evident through self-determination theory and digital platforms [23-30]; however, a significant gap remains in understanding the ROI, particularly when integrated into counseling-based EAPs. Studies suggest that peer support may lead to direct health care savings by equipping individuals with coping mechanisms and providing emotional support, which, in turn, reduces the risk of crises and costly subsequent interventions (eg, inpatient hospitalization, emergency room use) [31-33]. Specifically, current research does not adequately address how incorporating 24/7 DPS into EAPs translates into measurable cost-effectiveness for organizations. Traditionally, cost-effectiveness and cost-benefit analyses are used to assess the value for money of health and social interventions, but the value derived from participating in 24/7 DPS can be subtle and difficult to measure [34]. As a result, there is a paucity of research on the broader social, economic, and environmental value. This gap underscores the need for a focused study to evaluate the social return on investment (SROI) of integrating 24/7 DPS into a counseling-based EAP, assessing both the economic impact and the efficiency of these interventions.

### Social Return On Investment

SROI has become a recognized method for measuring the impact, outcomes, and value created by social-emotional–focused organizations [35,36]. Broadly, SROI uses a mixed-method design to assess the value of an intervention relative to the cost of enabling it. Beyond financial metrics, which may assign limited value to a program, SROI captures social, environmental, and economic elements to generate a comprehensive “social value.”

### Study Aim and Hypotheses

For this study, a 24/7 US-based DPS service was integrated with a US-based counseling-based EAP firm to explore potential changes in utilization, sentiment, and the SROI of the 24/7 DPS for the EAP’s clientele. The following study hypotheses were examined: (1) The introduction of the 24/7 DPS service may lead to changes in the utilization of EAP counseling services within a 5-session model; (2) participants who utilize the DPS service will experience significant changes in sentiment (eg, reduced sadness, loneliness, and stress) over the course of their engagement with the service; and (3) the integration of DPS into the EAP will produce a positive SROI, reflecting the added value of peer support services for EAP clientele. Through these key objectives, the study aims to assess the effects of DPS on EAP utilization, emotional well-being, and the social and economic value added to the EAP.

## Methods

### Pre- and Post-DPS Integration: EAP Services and Implementation

Using aggregated participant data, pre- and postanalyses of adding DPS to EAP services were conducted. Pre-DPS was defined as the routine care provided within the EAP, including an initial intake, screening for clinical concerns, and offering service options such as counseling, legal resources, psychoeducation, and coaching. Before the introduction of DPS, participants either accessed the EAP provider’s website using a company code to explore available services or completed an initial online or phone intake. They were screened for clinical concerns, including substance use and suicidal or homicidal ideation, by licensed clinicians or clinically supervised paraprofessionals. The EAP offers a comprehensive suite of behavioral health and wellness services designed to support mental health, work-life balance, and overall well-being. These offerings include free and confidential counseling, legal and financial benefits (including Medicare and Social Security consultation), wellness coaching, work-life referral services, and an online resource library. Additional services include virtual reality programs, as well as parenting, life, career, and work performance coaching. The EAP provides managed care 24/7, with crisis support delivered by licensed mental health professionals.

An anonymous US-based DPS service that provided 24/7/365 moderated, synchronous peer group chats was integrated into the US-based EAP’s client support ecosystem. The DPS service utilized artificial intelligence (AI)–driven natural language processing to match users with peers facing similar issues in small groups, each facilitated by a trained human moderator who ensured a safe environment and directed users to professional services in crises. The DPS model allows for unlimited sessions, in both duration and frequency, while the EAP’s 5-session model was selected for further analysis due to the prevalence of 5-session models over other session formats.

### Participants

#### DPS: Peer Support Users

Nonidentifying participant data from the DPS provider, collected between June 2023 and May 2024, were used to evaluate the research objectives. DPS service participants had access to unlimited peer support chats and were included in the data analysis if they utilized the chat service at least once.

#### EAP: Counseling Service Participants

##### Inclusion Criteria

Pre-DPS (June 2022 to May 2023) and post-DPS (June 2023 to May 2024) participants were selected based on having active access to the 5-session EAP model. For the analysis of total utilization, no demographic factors were matched between the pre- and post-DPS groups. However, for the utilization analysis of the 5-session EAP model and to ensure comparable groups, participants in both the pre-DPS and post-DPS cohorts were matched based on demographic and emotional concern variables. Matching was conducted using propensity score matching, adjusting for potential confounders such as age, gender, and emotional concerns (eg, anxiety or depression). Participants who had utilized the full 5-session model in both groups were selected, with efforts made to capture diverse client trajectories by including multiple participants matched on these same variables. EAP participants were employed across a wide range of sectors, including health care, education, professional services, manufacturing, construction, building trades, nonprofits, technology, service industries (eg, restaurants), unions, mining, government, and others.

##### Cohort Exclusion Criteria

Participants were excluded from the analysis if they had missing demographic data (age or gender) or if they exceeded the authorized number of counseling sessions (eg, >5 sessions in the pre-DPS group). These exclusions were necessary to control for confounding variables, as participants requiring more intensive mental health support (beyond 5 sessions) may have had significantly different needs compared with those who adhered to the 5-session model. This approach ensured that the study focused on participants whose engagement with EAP services reflected typical utilization patterns.

#### Peer Support Moderators

Peer support moderators (PSMs) are human moderators trained in digital safety monitoring who provide real-time, text-based support. PSMs receive up to 164 hours of training within the first 90 days of hire, focusing on engaging in digital conversations, facilitating synchronous group chats, and managing the psychological safety of chat users by monitoring for and removing trolls, as well as enacting safety protocols, such as referring users who may be in active crisis to appropriate services. Additionally, PSMs receive 36 hours of ongoing asynchronous and synchronous clinical supervision and consultation per quarter.

### Procedures

#### Overview

This retrospective study evaluated the differences in the EAP’s services before and after DPS to assess the programmatic impact and added social value.

#### Data Variables

The primary data sources for this study were aggregated engagement variables, such as chat time, number of chats, age, gender, user struggles or presenting concerns, and national-level cost-of-service value data.

#### Data Extraction and Management

The data extraction process involved retrieving DPS chat data, which included digital chat records, user interactions with the platform, and usage patterns. All collected data were deidentified and anonymized to ensure participant privacy and confidentiality. Data management procedures adhered to institutional and regulatory guidelines, ensuring data security and integrity, with access restricted to authorized personnel. Measures were in place to maintain data security, integrity, and confidentiality throughout the analysis process.

### Statistical Analysis

#### Analysis and Group Comparability

Descriptive statistics were used to summarize participant characteristics and their engagement with DPS and EAP services. To assess differences in service utilization and sentiment scores between pre-DPS and post-DPS groups, paired *t* tests (2-tailed) were conducted for continuous data (eg, session frequency), and chi-square tests were used for categorical data (eg, gender, emotional concern). As DPS is anonymous, it was not possible to directly track which EAP participants used the service. Using volunteered age and gender data from DPS participants as a starting point, propensity score matching was applied to control for potential confounders such as age, gender, and emotional concern, ensuring group comparability before DPS integration.

#### Mood Categorization

Clinical counseling experts reviewed the emotional concerns reported by DPS participants, categorizing them into mood categories such as depression, anxiety, adjustment, and relational concerns to align DPS participants with the presenting emotional concerns of EAP participants. The counseling experts were 2 licensed psychologists with doctoral degrees in counseling psychology and a combined 50 years of experience in the field. The clinical interpretation process, guided by the Biopsychosocial Model [37], involved categorizing participants’ emotional struggles into mood categories. This model integrates biological, psychological, and social dimensions to provide a comprehensive understanding of mental health. For example, participants’ reports of feeling “trapped” or “isolated” were examined through a psychological lens to identify depressive thought patterns, while work-related stress (eg, “I hate my boss”) was contextualized within the social environment to classify relational concerns [38]. By incorporating these factors, the model enabled clinical experts to holistically interpret participants’ struggles, ensuring that the categorizations captured the complexity of their emotional experiences. The categorization process involved initially reviewing the raw data from participants’ reported “struggles” and presenting concerns in therapy individually, followed by cross-validation between the 2 experts to ensure consistency. This collaborative step helped reconcile differences in categorization and address overlapping cases. It is important to note that neither the text-based struggle data from DPS nor the presenting concerns from EAP represented formal diagnoses; rather, they reflected participants’ personal interpretations of their symptoms. Keywords such as *helpless* or *sadness* were flagged for depression-related presenting concerns, while terms related to *fear, excessive worry,* or *panic* were matched with anxiety-focused concerns. In cases where symptoms overlapped (eg, depression and anxiety), the experts aligned their categorizations based on the primary presenting concern reflected in the participant’s statement. For example, if a participant’s statement indicated feelings of hopelessness and despair alongside anxious thoughts, the struggle was categorized as depression. Alternatively, if worry or nervousness predominated, it was categorized as anxiety. In scenarios where a specific relationship was mentioned alongside anxious or depressive feelings, the struggle was categorized primarily as a relational concern. Symptom overlap was resolved by categorizing the statement according to the dominant theme present in the participant’s experience. Table 1 provides examples of these interpretations.

**Table 1:** Clinical interpretation of comparable emotional concerns shared on digital peer support service with employee assistance program service.

Participant’s struggle	Presenting concern^a^
“I am feeling pretty suicidal and trapped. I am new to my work from home job and I need help, resources, a plan something or this will only get worse. I am trapped with no transportation, no friends or any meaningful relationships in life. Just a work schedule and I’m isolated.”	Depression
“I hate my job and my boss”	Relational concern
“I’ve been feeling a lot of anxiety around work and my personal life”	Anxiety/stress
“Alcohol addiction”	Substance use

^a^These are not diagnoses, they are layperson understandings.

### Sentiment Analysis

Peer-to-peer conversations were analyzed to produce a quantitative measure of emotional change. Each participant’s chat session, referred to as a “user-chat-session” or simply “user-chat,” was treated as an independent unit of analysis, regardless of whether the same participant took part in multiple sessions. As the DPS service is anonymous, each user and their experience in a given chat session were considered separately, without combining data from different sessions for the same user. This approach ensures that each user’s experience is analyzed in isolation, providing a more accurate, user-centric view of emotional changes.

Each user’s message was analyzed in the context of preceding messages from other users to capture their emotional journey throughout the chat session. Context was defined as the concatenation of all preceding messages from other users in the same chat, starting from the latest message of the user of interest. A user’s initial struggle and first message had no prior context and were evaluated with an empty context. For example, in the following conversation:

userA: message1

userB: message2

moderator: message3

userA: message4

userB: message5

This example assumes that message1 and message2 are the first messages of user A and user B, respectively, in the chat session. User A’s emotional journey was quantified by evaluating message1 with an empty context and message4 in the context of message2 + message3. Similarly, for user B, message2 was evaluated without context, and message5 was evaluated in the context of message3 + message4.

Each user’s message, along with its corresponding context, was evaluated for sentiments of sadness, loneliness, and stress using a scale of 1-10, where 1 indicates low intensity, 5 indicates moderate intensity, and 10 indicates high intensity of the sentiment. The analysis focused on chat sessions where participants exhibited at least moderate sentiment intensity (score≥5) from the beginning.

A third-party, public natural language processing model from OpenAI, called GPT-4, was used to assign sentiment scores to each user message and its preceding context [39]. A few-shot learning approach was applied, where the model was provided with 10 examples representing different sentiment intensities to calibrate its interpretations [40]. GPT-4 demonstrates an advanced capacity to interpret emotion, surpassing benchmarks from the general population and offering highly congruent performance in emotion interpretation [41]. See Table 2 for examples provided to GPT-4 to predict loneliness levels.

Sentiment analysis evaluated each user’s chat messages in temporal order. The quantified emotions were then interpolated and extrapolated to generate a collective sentiment trend across peer-to-peer chats of varying lengths. A one-sided Mann-Whitney *U* test was used to compare emotion scores at the start and end of the conversations.

**Table 2:** Examples provided to GPT-4 to interpret loneliness intensities in a few-shot manner.

Intensities	Loneliness
1=low	“Take care everyone. Thanks for the great talk”
5=moderate	“I went on Monday and today. After today's class is when I felt worse as I chatted with a lady in class who told me that she got really depressed when she retired.”
10=high	“I just always feel totally alone. Like no one understands what is going on in my head”

### SROI Methodology

Historically, the SROI methodology has relied on qualitative methods to identify outcome themes most relevant to participants, followed by quantitative approaches to determine monetary proxies for the identified outcomes and related values [41].

With the advent of natural language processing models, significantly larger volumes of text-based data can now be analyzed, allowing qualitative data to be graphically represented. This study utilized text-based data from peer group participants—including open-ended questions, affirmations, reflective questions, and summaries—to examine changes in sentiments such as sadness, loneliness, and stress within peer-support chats [42]. The SROI methodology was applied to assess the social value created by integrating DPS into the EAP. Qualitative data from peer-to-peer conversations, including open-ended questions, affirmations, and summaries, were analyzed to understand sentiment changes and emotional shifts within the peer group chats. Quantitative methods were used to calculate a monetary proxy for the emotional outcomes. The Sopact SROI calculator, available on the Sopact website [41], was utilized to determine the social value generated by the DPS service, incorporating factors such as enhanced emotional well-being and cost savings from reduced therapy usage. Given the novelty of DPS and its integration into the EAP, a small sample size was anticipated. This limitation was addressed through rigorous matching techniques and statistical analyses to ensure the findings remained valid and robust despite the smaller sample size. Moreover, it is acknowledged that some participants may have preferred individual counseling services over DPS, potentially introducing selection bias among those who opted for the DPS model.

### Ethical Considerations

Informed consent was obtained from all participants before their involvement in the study. The consent process included clear explanations of the research’s purpose, data collection methods, and the measures implemented to ensure participant privacy and confidentiality. All data collected were deidentified to safeguard participants’ anonymity. The study complied with all institutional and regulatory guidelines related to data protection and participant privacy. To ensure participant safety within the DPS, real-time moderation was provided by trained human moderators specializing in digital safety monitoring. Furthermore, the Pearl Institutional Review Board reviewed and approved the study (approval ID 2024-0442).

## Results

### Total DPS Utilization

- Study hypothesis 1: The introduction of the 24/7 DPS service may be associated with utilization changes of EAP counseling services within a 5-session model, specifically among participants who engage with DPS.

Out of 587 total chats, 432 took place between 5 PM and 8 AM, hours when most therapy offices are typically closed. Chats lasting less than 5 minutes were excluded from the analysis, as the initial 5 minutes are generally used for introductions and setting expectations [30]. Consequently, 107 chats were omitted. A summary of the data is provided in Table 3.

**Figure 1:**
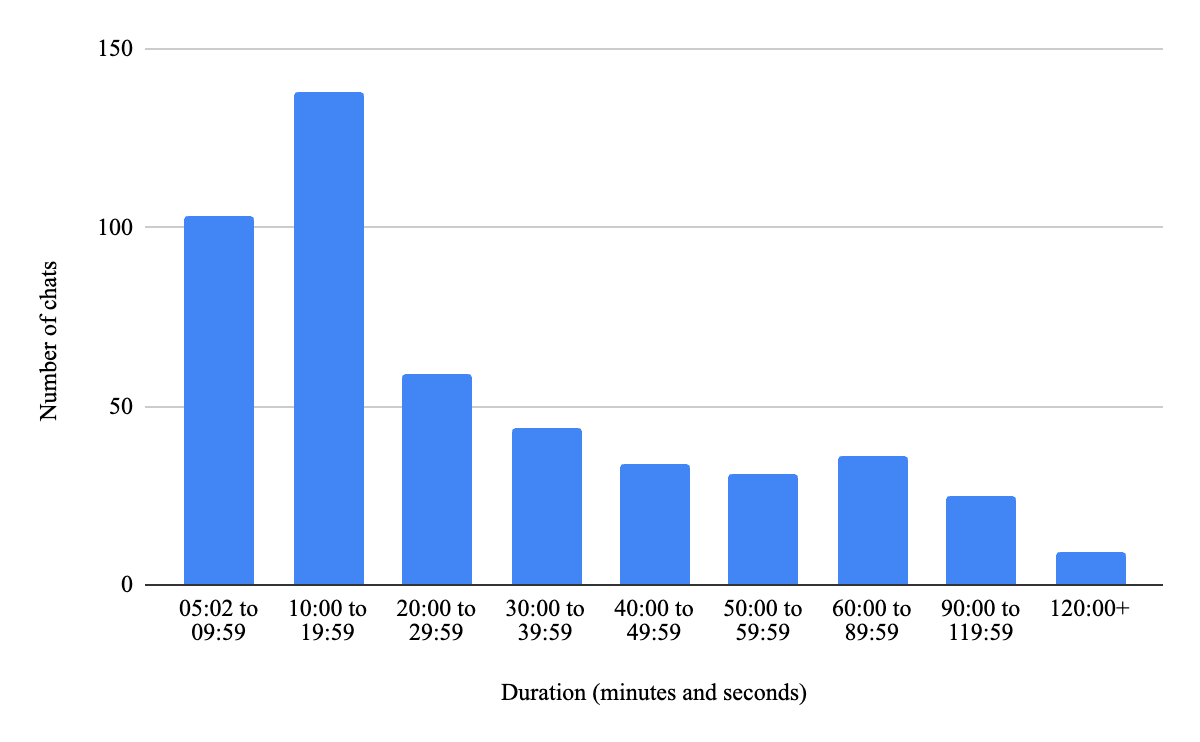
Chat duration distribution.

When categorized by duration, the data revealed that 103 chats lasted between 0 and 9 minutes 59 seconds, 138 chats between 10 and 19 minutes and 59 seconds, and 59 chats between 20 and 29 minutes and 59 seconds. Additionally, 44 chats lasted between 30 and 39 minutes and 59 seconds, 34 chats between 40 and 49 minutes and 59 seconds, and 31 chats between 50 and 59 minutes and 59 seconds. While longer chats were less common, they occurred consistently: 36 chats lasted between 60 and 89 minutes and 59 seconds, 25 chats between 90 and 119 minutes and 59 seconds, and 9 chats exceeded 120 minutes. Refer to Figure 1 for further details.

**Table 3:** Peer support program utilization data.

Metric	Value
Total chats, n	480
Total messages, n	7219
Total duration, hours:minutes:seconds	254:17:57
Average duration, hours:minutes:seconds	0:31:51
Shortest duration, hours:minutes:seconds	0:05:02
Longest duration, hours:minutes:seconds	3:19:11

### Pre-Post Analysis

#### Matched Cohort Analysis

To evaluate the utilization impact of DPS on the EAP’s 5-session model, semimatched cohorts were created for the periods before (June 2022 to May 2023) and after DPS implementation (June 2023 to May 2024). Because of the anonymous nature of the DPS service, only participants who voluntarily provided their age and gender were included in the matching process for analysis in the EAP’s pre- and post-DPS cohorts. The matching variables comprised age, gender, and presenting emotional concerns.

#### Sample

Data on presenting emotional concerns were readily available based on participants’ responses to the question, “What’s your struggle?”—the sole query asked before matching them with the DPS service. A total of 45 unique users provided both their age and gender information voluntarily. These users ranged in age from 18 to 51 years, with a mean age of 34.97 (SD 9.71) years. Gender distribution was as follows: 18 (40%) males, 26 (58%) females, and 1 (2%) nonbinary individual.

The availability of participant age, gender, and emotional concern data from the DPS service enabled the creation of a matched pre-DPS EAP cohort. The frequency of emotional concerns in the peer support program sample is presented in Table 4.

**Table 4:** Emotional concern frequency in the digital peer support sample (n=45).

Emotional concern type	Value, *n* (%)
Adjustment	1 (2)
Depression	18 (40)
Anxiety	8 (18)
Posttraumatic stress disorder	2 (4)
Relational concern	14 (31)
Substance use	2 (4)

#### Statistical Analysis

Using age and gender data from the DPS service participant pool, a total sample of 72 EAP participants—36 pre-DPS and 36 post-DPS—was analyzed for differences. The ages of EAP participants ranged from 18 to 51 years, with a mean age of 33.86 (SD 8.18) years. Females comprised the majority of participants (n=68, 94%), while males accounted for a smaller proportion (n=4, 6%) in both the pre- and post-DPS cohorts.

To assess the impact of the DPS addition, a paired samples *t* test (2-tailed) was performed, comparing the number of therapy sessions utilized by participants pre- and post-DPS within the matched cohort. The analysis revealed a mean difference of –2.07 (SD 1.77) sessions (*t*_71_=9.92; *P*<.001), indicating a significant reduction in the number of therapy sessions used after the intervention. These findings suggest that the intervention may have been associated with a decrease in therapy session utilization among the sampled participants.

### Effect Size

In addition to the *t* test, Cohen *d* was calculated to determine the effect size of the intervention. Based on Cohen guidelines, effect sizes are classified as small (0.2), medium (0.5), and large (0.8). The effect size in this study was 1.77, which is considered large. This suggests that the DPS service had a substantial impact on reducing the number of therapy sessions utilized by participants.

The CI for the mean difference in therapy session utilization was calculated to be between –1.47 and –0.87 at the 95% CI level. This range further supports the reliability of the observed reduction in session utilization, providing evidence that the DPS service may have effectively decreased the number of therapy sessions used by participants in the small sample group.

- Study hypothesis 2: Participants who utilize the DPS service will experience significant changes in sentiment (eg, reduced sadness, loneliness, and stress) throughout their engagement with the service.

### Sentiment Change

**Figure 2:**
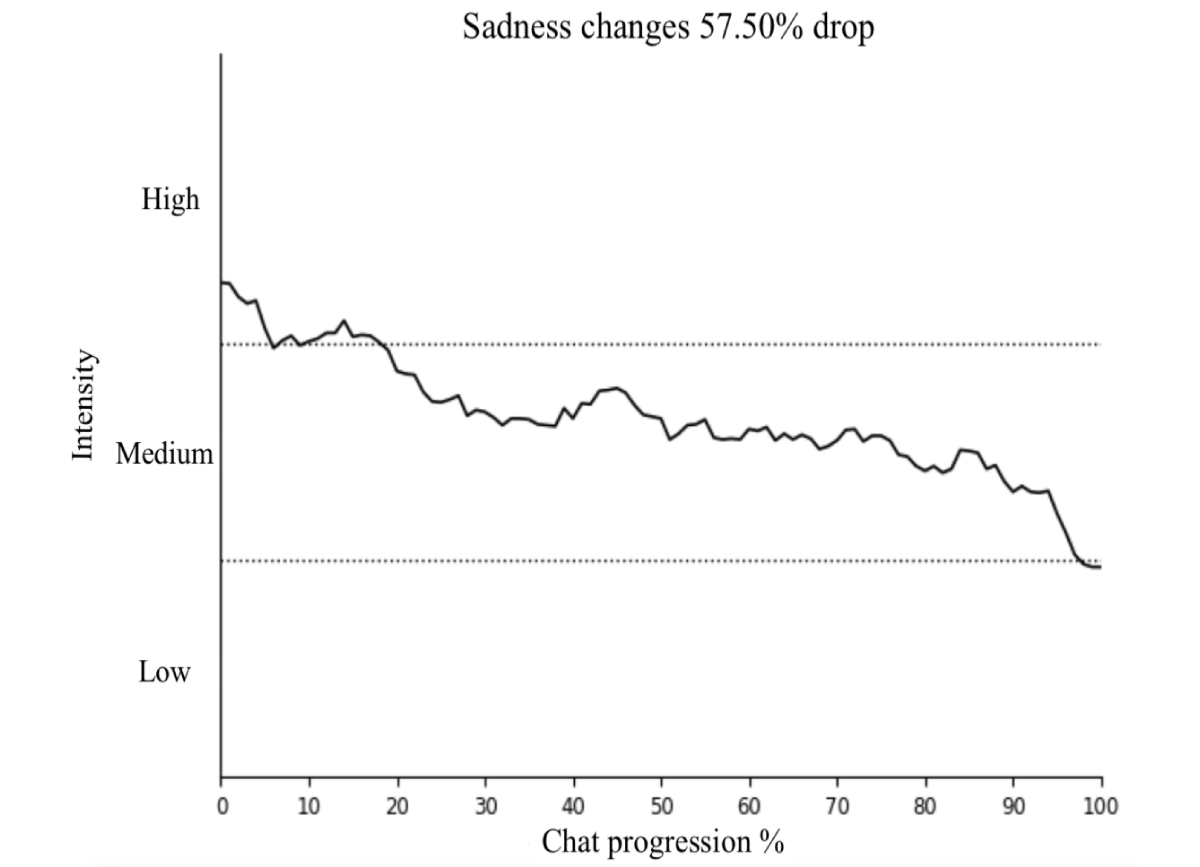
Significant (*P*<.001) changes in sadness sentiment during chats.

**Figure 3:**
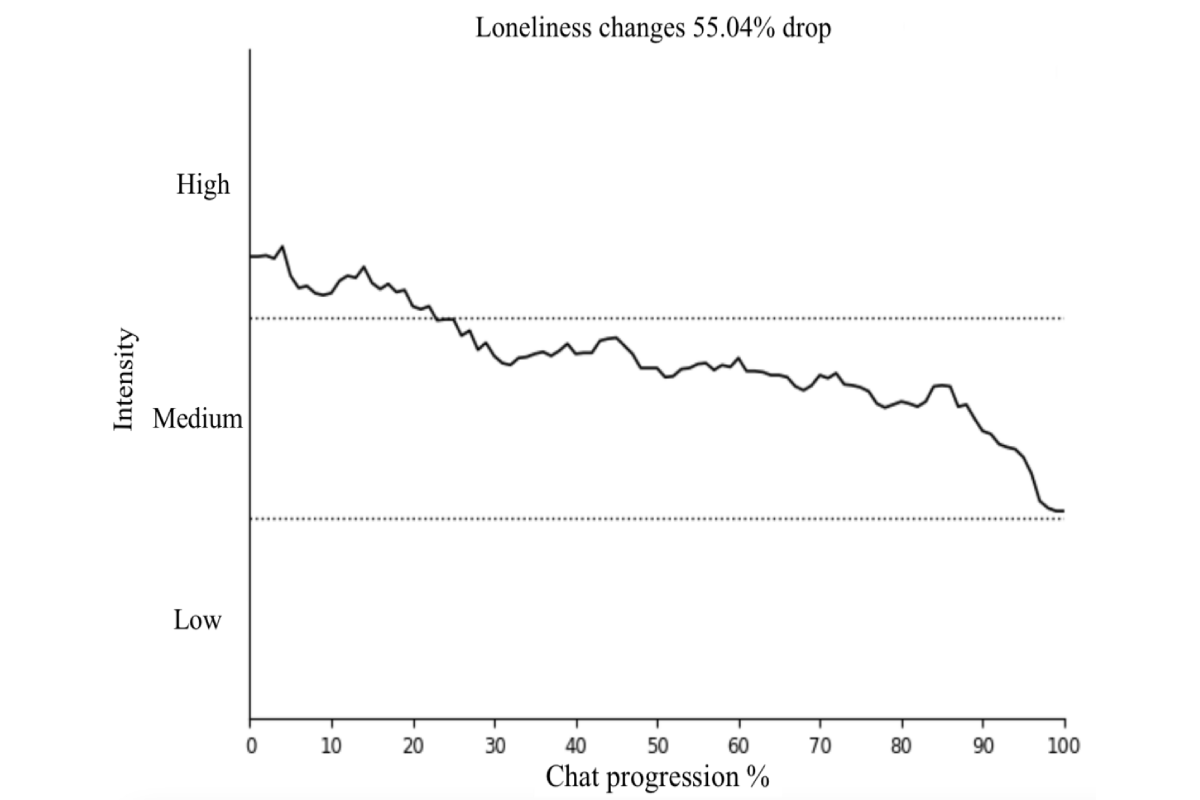
Significant (*P*<.001) changes in loneliness sentiment during chats.

**Figure 4:**
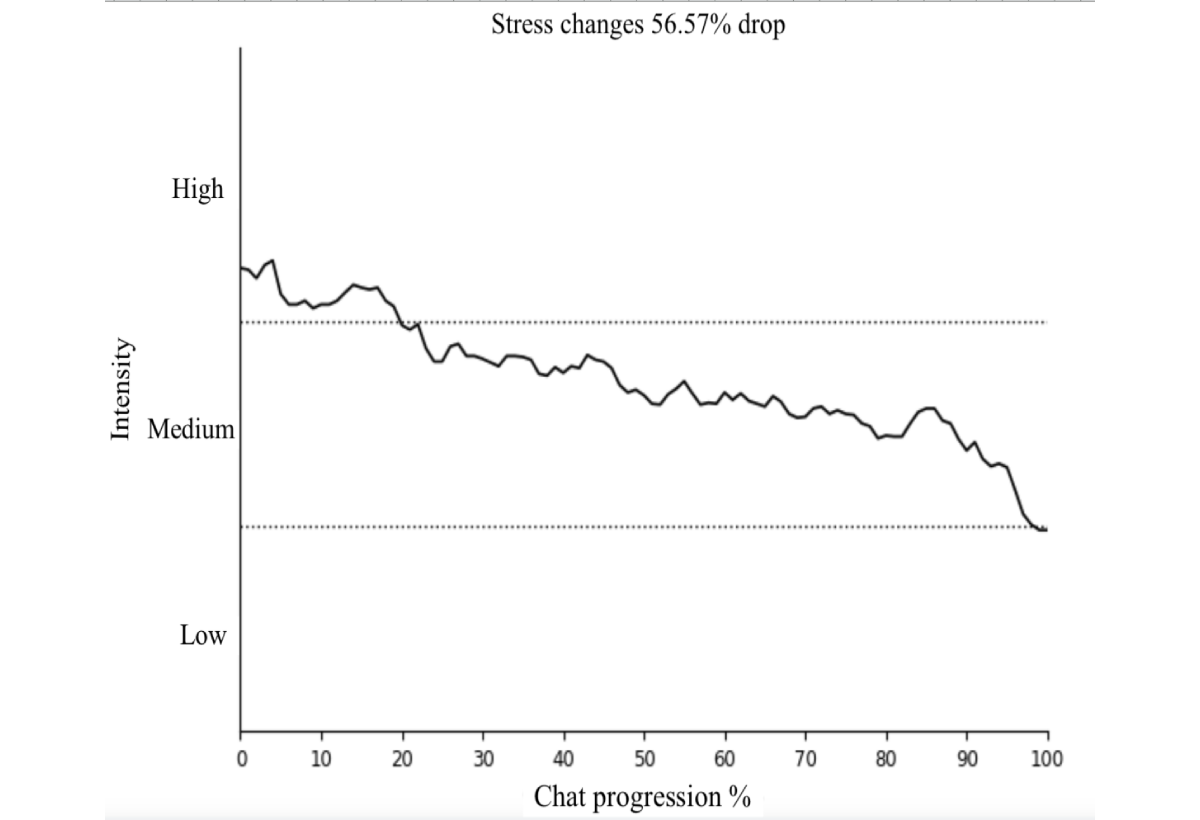
Significant (*P*<.001) changes in stress sentiment during chats.

From the DPS service’s sampled cohort (n=45), sentiment changes for sadness, loneliness, and stress were investigated for each user chat. The results are presented in Figures 2-4, showing the changes in sentiment for these 3 emotions. All 3 emotions demonstrated statistically significant reductions (sadness, loneliness, and stress: *P*<.001) in sentiment intensity during the chat sessions. The sentiment intensity levels are categorized as follows: 1-4 as “low,” 4-7 as “moderate,” and 7-10 as “high.”

### Social Return on Investment Analysis

SROI assigns financial proxies to quantify the value of each identified sentiment change, enabling the attribution of a market price when no direct market value exists [41]. The proxies were carefully selected based on their relevance and supported by cited research studies to ensure the credibility of the assigned market prices. Table 5 presents the financial proxies used for each sentiment.

- Study hypothesis 3: The integration of DPS into the EAP will generate a positive SROI, reflecting the added value of peer support services for EAP clientele.

**Table 5:** Financial proxies for each sentiment.

Factors in which digital peer support users experience a reduction	Financial proxy	Cost
Sadness	Average unit cost of treating someone with depression (National Institutes of Health)	PPPY^a^: US $10,074 (SD $25,694) PPPM^b,c^: US $839.50
Loneliness and isolation	Average unit cost of treating someone with objective isolation and loneliness (National Institutes of Health)	PPPY: US $1643 in Medicare spending (objective isolation) PPPM: US $136.92
Stress (eg, generalized anxiety)	Average unit cost of treating someone with stress	PPPY: US $6475 PPPM: US $539.58

^a^PPPY: per person per year.

^b^PPPM: per person per month.

^c^PPPM calculations were made by dividing the PPPY value by 12 months.

### SROI Analysis

The primary objective of this SROI analysis is to calculate and present a ratio that compares the cost of investment (US $) in an intervention with the total social, environmental, and economic value (US $) it generates. To prevent overestimation, the SROI methodology recommends certain considerations (see Table 6) [41].

The SROI analysis demonstrated that the DPS program generates positive social value, complementing overall treatment. The SROI values ranged from US $1.66 (loneliness) to US $2.50 (stress) to US $2.58 (sadness) for every dollar invested. Detailed calculations for the impact ratio are available in Multimedia Appendix 1.

**Table 6:** Factors considered for calculating the social return on investment impact ratio.

Factor	Description
Deadweight	A measure to describe the amount of an outcome that would have happened anyway, even if the DPS^a^ service had not been offered. For example, the participants experiencing reductions in momentary loneliness may have a deadweight value of 10% from simply joining the DPS chat, but without further intervention from the peer support moderator or other peers.
Displacement	An assessment of what activities or services are displaced by the presence of the digital peer group. There was no evidence of displacement as, before the digital support option was added, there were no after-hours DPS chat services for employee assistance program members to access.
Attribution	A measure to consider how much of an identified theme is a result of the group studied or is influenced by external factors, for example, if participants also attend another service, such as counseling, which reduces their sense of chronic sadness, a conservative estimate of 20% of the identified momentary sentiment change may be attributed to the group studied.
Dropoff	The percentage of the outcome that decreases after the intervention is complete. For example, a conservative percentage of 10% re-emergence of stress may be expected after the user ends their DPS chat due to a return to their stressful environment.

^a^DPS: digital peer support.

## Discussion

### Principal Findings

The study examined the impact of integrating a DPS service within an EAP on utilization rates, sentiment change, and therapy session use over 1 year. Additionally, it assessed the social, environmental, and economic impact of the DPS service by estimating an SROI value. Results indicated that DPS service utilization primarily occurred after hours. In the matched sample, participants used fewer therapy sessions post-DPS integration. The SROI ranged from US $1.66 to US $2.50 to US $2.58 for every dollar invested for loneliness, stress, and sadness sentiments, respectively. These findings suggest that combining DPS with traditional EAP services may provide noncrisis emotional support after hours, potentially reducing EAP therapy session use for lower-acuity emotional concerns. This, in turn, could allow EAPs to allocate funds toward additional ancillary support services and enable employers to benefit from a positive SROI.

The study’s findings—that participants experience significant improvements in sentiment, such as reduced sadness, loneliness, and stress—align with existing literature on patient activation [7,8]. Research indicates that when individuals are activated—meaning they gain confidence and skills in managing their health—they tend to experience improvements in emotional well-being [8]. By providing peer support that fosters autonomy and self-efficacy, participants can develop coping strategies that reduce distress and promote mental health [43]. This aligns with findings that patient activation leads to better emotional outcomes through the development of personal coping mechanisms and self-management [8]. The positive SROI from integrating DPS into EAP services is also consistent with the literature on patient activation. Activated patients, who feel more in control of their health, contribute to more efficient resource utilization and improved outcomes [44]. The integration of peer support services such as DPS has been shown to enhance engagement with formal mental health services, creating value not only for individuals but also for the broader service system [44]. This supports the idea that empowering individuals through peer support can yield both personal and systemic benefits, ultimately leading to a positive SROI.

The study may also contribute to the quality of life literature, which is commonly used in cost-effectiveness studies. Health-related quality of life (HRQoL) refers to a person’s physical, emotional, and social well-being as influenced by a health intervention [45]. While this study did not include standardized HRQoL assessments (eg, 36-item Short Form [SF-36] or EQ-5D) due to real-world data collection constraints and the anonymous nature of the DPS service, sentiment changes and therapy utilization could serve as HRQoL proxies [46]. Significant reductions in sadness, loneliness, and stress during DPS chats suggest improvements in emotional well-being, a core component of HRQoL. The reduction in momentary negative sentiments may indicate that participants used DPS as a coping mechanism for emotional stability. Additionally, the suggested decrease in therapy sessions implies that participants may have achieved better emotional regulation or relief through DPS, ultimately impacting their overall quality of life. Relatedly, the high utilization of DPS after hours addresses a gap in traditional mental health services and may contribute to participants’ perceived control over their emotional health. Such improvements in HRQoL proxies could translate into better job performance [47] and employee satisfaction [48], while also serving as a valuable complement to therapy. This approach allows mental health providers to focus on higher-acuity cases while DPS services address lower-acuity concerns—key objectives for most EAP organizations [48]. Finally, the calculated SROI highlights tangible benefits for emotional health (eg, sadness, loneliness, stress), further demonstrating the intervention’s effectiveness in enhancing HRQoL’s socioemotional components.

The study’s findings further align with existing literature in several key areas. The use of AI for matching participants with peers who have shared lived experiences, as well as for sentiment analysis, supports growing evidence that AI can enhance the personalization and efficiency of life struggle and nonmental health services [49-52] This study offers several novel contributions to the literature on mental health interventions and EAPs. It is among the first to demonstrate that integrating a DPS service with EAPs may enhance after-hours support, reduce the utilization of EAP therapy sessions—allowing EAPs to allocate resources more effectively for higher-acuity emotional concerns—and provide supplementary SROI. DPS services are unique within the EAP environment and may be particularly valuable for EAP participants who are not yet ready to confront the stigma or commitment often associated with therapy [53] but still benefit from peer support. By leveraging AI-driven natural language processing to match users based on their struggles and conducting sentiment analysis on chat narratives, this study highlights an innovative use of technology to enhance support for stress and life challenges. Conducted in a naturalistic setting with a diverse sample, the study’s design strengthens ecological validity, demonstrating the practical feasibility and effectiveness of DPS in real-world organizational environments.

### Study Limitations

This study faced several potential threats to both internal and external validity and implemented strategies to mitigate these issues. One threat to internal validity was the influence of external events (history) during the study period (June 2023 to May 2024) [54,55]. To address this, a comparison group from the previous year (June 2022 to May 2023) was used, and quarterly data monitoring helped identify and adjust for significant external changes [55]. Matched samples based on additional demographics were not possible due to the anonymity of DPS users. Future research using alternative study designs with nonanonymized data may be necessary.

Maturation effects, referring to the natural changes in participants’ mental health over time, were addressed using a cohort design with propensity score matching. This approach ensured that participants in both the intervention and control groups were similar in age, gender, and presenting concerns [56]. By balancing the groups, it minimized the impact of maturation on the results [56]. Additionally, selection bias was mitigated through propensity score matching, which created comparable groups by matching participants in the pre-DPS and post-DPS periods based on key demographics and presenting concerns [57].

Experimental mortality, or dropout, was another concern. To address this, the study included multiple participants matched on the same variables, ensuring that the analysis could proceed even if some participants dropped out [57]. A diverse sample across age and gender was included to reduce the impact of selection biases on generalizability. Additionally, conducting the study in a naturalistic setting, where participants used the DPS and EAP services as they would in real life, helped minimize the reactive effects of experimental arrangements, enhancing ecological validity [55]. Furthermore, the availability and normalization of the EAP program may have influenced participant engagement. EAP programs are voluntary for companies in the United States, Australia, Canada, and England, whereas Nordic countries have legal mandates requiring mandatory EAPs [58]. This distinction is relevant from the perspective of patient activation. Voluntary EAPs may require high awareness and active promotion to engage participants and may primarily attract individuals with higher activation or those who are more self-directed. As a result, individuals with lower activation or those in the early stages of their mental health concerns may remain underserved [58]. Additionally, factors such as program promotion and mental health stigma could contribute to uneven access across the workforce. By contrast, mandatory EAPs may help normalize access to mental health support, encourage early and preventive engagement, and promote continuity of care by integrating with public health systems, unlike the segmentation often seen with voluntary EAPs [59]. Limitations of the SROI process include the complexity of assigning financial proxies to sentiment change and the availability of data required for robust calculations, such as displacement and attribution values. A key risk in SROI analysis is an overemphasis on the ratio itself, without considering the underlying content, which provides deeper insight into the value created by different groups [58]. By acknowledging and addressing these challenges through design and analysis strategies, the study generated meaningful insights into the impact of integrating DPS services within an EAP.

### Future Research

The study opens several avenues for future research. Long-term impact assessments are needed to evaluate how integrating DPS with EAP services influences therapy utilization and outcomes over extended periods. A broader demographic analysis could provide insights into how factors such as age, socioeconomic status, and cultural background affect engagement with these services. Additionally, incorporating qualitative insights may help illuminate user experiences and satisfaction, offering a deeper understanding of which aspects of the service are most effective or subjectively valued by clients.

Future research could also compare DPS with other digital mental health interventions to identify which models offer the greatest benefits in different contexts. Investigating how DPS integrates with other mental health services, such as primary care or community programs, could provide a more comprehensive understanding of its role within the broader health care system. While comparisons of single- versus multilayered support ecosystems fall beyond the scope of this study, they represent a valuable avenue for future exploration (eg, digital mental health vs in-person services or EAP vs DPS). Additionally, examining DPS’s impact on different mental health conditions and conducting a detailed economic analysis of cost savings could further strengthen the case for its adoption. Moreover, this study did not calculate quality-adjusted life years; however, the observed improvements and associated cost reductions provide strong support for the intervention’s economic value. Future research could explore additional methods to further quantify these benefits. These efforts would deepen our understanding of DPS’ effectiveness and its potential applications across diverse settings.

### Conclusions

This study demonstrates that integrating DPS services within an EAP may influence therapy utilization, allowing EAPs to serve higher-acuity clients while lower-acuity clients access supplementary support, such as DPS, for everyday emotional needs. Additionally, the research highlights the potential social, environmental, and broader economic benefits of incorporating DPS services into the emotional support ecosystem, with positive estimated SROI outcomes. By leveraging AI-driven natural language processing for user matching and sentiment analysis, the study underscores the potential of technology to enhance stress management, address life challenges, support mental health, and optimize resource allocation. The findings emphasize the need for further research into DPS effectiveness, particularly regarding long-term impacts, demographic variations, and comparative effectiveness with other interventions. Overall, this study provides valuable insights into the evolving landscape of digital mental health services, reinforcing their role in enhancing organizational wellness programs and identifying key areas for future exploration to maximize their potential.

## Appendix

Multimedia Appendix 1 Breakdown of SROI calculations as calculated using the SROI calculator available on the Sopact website. SROI: social return on investment.

## Abbreviations

AI: artificial intelligence
DPS: digital peer support
EAP: employee assistance program
HRQoL: health-related quality of life
PSM: peer support moderator
ROI: return on investment
SF-36: 36-item Short Form
SROI: social return on investment
