# Supplementary material for: Exploring the Impact of Digital Peer Support Services on Meeting Unmet Needs Within an Employee Assistance Program: Retrospective Cohort Study
Source: JMIR Hum Factors. 2025 Feb 25;12:e68221. doi: 10.2196/68221 (PMC11897672; doi:10.2196/68221)
Supplement: Multimedia Appendix 1 [file humanfactors_v12i1e68221_app1.docx]

## Appendix 1

*Table S1: Breakdown of SROI calculations as calculated using the SROI calculator available on SOPACT.com.*

| Sentiment | PPPY ($) | PPPM ($) | Total Investment | Quantity | Deadweight | Displacement | Attribution | Dropoff | Calculation | SROI Results |
| --- | --- | --- | --- | --- | --- | --- | --- | --- | --- | --- |
| Sadness | $10,074 | 839.50 | 50,000 | 1000 | 10 | 5 | 20 | 10 | * Total Value of Outcomes = 1000 * $839.50 = $839500.00  * Net Value = $839500.00 * (1 - 0.10) * (1 - 0.05) * 0.20 * (1 - 0.10) = $129199.05  * SROI Ratio = $129199.05 / $50000.00 = 2.58 | Total Outcome: $839500.00  Net Value: $129199.05  SROI Ratio: 2.58 |
| Stress | $6475 | 539.58 | 50,000 | 1000 | 10 | 5 | 20 | 10 | * Total Value of Outcomes = 1000 * $539.58 = $539580.00  * Net Value = $539580.00 * (1 - 0.10) * (1 - 0.05) * 0.20 * (1 - 0.10) = $83041.36  * SROI Ratio = $83041.36 / $50000.00 = 1.66 | Total Outcome: $539580.00  Net Value: $83041.36  SROI Ratio: 1.66 |
| Loneliness & Isolation | $1643 | 136.92 | 50,000 | 5930 | 10 | 5 | 20 | 10 | * Total Value of Outcomes = 5930 * $136.92 = $811935.60  * Net Value = $811935.60 * (1 - 0.10) * (1 - 0.05) * 0.20 * (1 - 0.10) = $124956.89  * SROI Ratio = $124956.89 / $50000.00 = 2.50 | Total Outcome: $811935.60  Net Value: $124956.89  SROI Ratio: 2.50 |
